# Solution/Ammonolysis Syntheses of Unsupported and Silica-Supported Copper(I) Nitride Nanostructures from Oxidic Precursors

**DOI:** 10.3390/molecules26164926

**Published:** 2021-08-14

**Authors:** Robert Szczęsny, Tuan K. A. Hoang, Liliana Dobrzańska, Duncan H. Gregory

**Affiliations:** 1 WestCHEM, School of Chemistry, University of Glasgow, Glasgow G12 8QQ, UK; Hoang.Tuan@hydroquebec.com; 2 Faculty of Chemistry, Nicolaus Copernicus University in Toruń, Gagarina 7, 87-100 Toruń, Poland; lianger@umk.pl; 3 Hydro-Québec Research Institute, Hydro-Québec, 1806, Boul. Lionel-Boulet, Varennes, QC J3X 1S1, Canada

**Keywords:** copper nitride, ammonolysis reaction, urea, CuO, Cu(OH)_2_, Cu_2_(OH)_3_Cl

## Abstract

Herein we describe an alternative strategy to achieve the preparation of nanoscale Cu_3_N. Copper(II) oxide/hydroxide nanopowder precursors were successfully fabricated by solution methods. Ammonolysis of the oxidic precursors can be achieved essentially pseudomorphically to produce either unsupported or supported nanoparticles of the nitride. Hence, Cu_3_N particles with diverse morphologies were synthesized from oxygen-containing precursors in two-step processes combining solvothermal and solid−gas ammonolysis stages. The single-phase hydroxochloride precursor, Cu_2_(OH)_3_Cl was prepared by solution-state synthesis from CuCl_2_·2H_2_O and urea, crystallising with the atacamite structure. Alternative precursors, CuO and Cu(OH)_2_, were obtained after subsequent treatment of Cu_2_(OH)_3_Cl with NaOH solution. Cu_3_N, in the form of micro- and nanorods, was the sole product formed from ammonolysis using either CuO or Cu(OH)_2_. Conversely, the ammonolysis of dicopper trihydroxide chloride resulted in two-phase mixtures of Cu_3_N and the monoamine, Cu(NH_3_)Cl under similar experimental conditions. Importantly, this pathway is applicable to afford composite materials by incorporating substrates or matrices that are resistant to ammoniation at relatively low temperatures (ca. 300 °C). We present preliminary evidence that Cu_3_N/SiO_2_ nanocomposites (up to ca. 5 wt.% Cu_3_N supported on SiO_2_) could be prepared from CuCl_2_·2H_2_O and urea starting materials following similar reaction steps. Evidence suggests that in this case Cu_3_N nanoparticles are confined within the porous SiO_2_ matrix.

## 1. Introduction

Copper nitride is nontoxic and relatively stable at ambient conditions. It is a semiconductor with high electrical resistivity, low reflectivity, and low thermal stability [1,2,3]. With regard to its potential commercial value and interesting properties, Cu_3_N has been exploited for applications in optical storage media [4,5,6,7,8], as a component of spintronic systems [9,10], as an electrode material in rechargeable Li- and Na-ion batteries [11,12,13,14] and as a highly active catalyst [15,16,17,18,19]. More specifically, thin films of Cu_3_N can be utilized in the fabrication of microscopic metal lines, dots, or Cu/Cu_3_N microscopic structures using electron beam or laser irradiation [7,20,21,22,23,24]. These processes can be implemented effectively by exploiting the inherent thermal instability of copper nitride. Thermal treatment at a moderate temperature decomposes Cu_3_N into nitrogen and pure metallic copper and the deposited Cu metal unsurprisingly has a significantly higher reflectivity and conductivity than its nitride. Otherwise, the magnetic properties of Cu_3_N offer applicability in devices as spin valves [7] or can be exploited to examine the spin excitations of antiferromagnetic Mn chains [6]. Cu_3_N forms a cubic anti-ReO_3_ type structure (space group *Pm*-3*m*) where nitrogen atoms are located at the corners of the primitive unit cell and copper atoms occupy the 3*d* edge sites. Given the open nature of the structure, in some circumstances, it is possible to insert metal atoms into the interstitial body centre (½,½,½) position [25,26]. According to previous studies, the addition of a transition metal modifies not only the electronic properties of Cu_3_N but also its magnetic characteristics and, for example, provides a route to magnetic semiconductors [3,27].

Whereas films of copper nitride have primarily been fabricated by physical methods such as reactive RF magnetron sputtering [28,29,30] or pulsed laser deposition (PLD) [31,32], bulk Cu_3_N was first synthesized by Juza and Rabenau many decades before by heating a CuF_2_ precursor under gaseous ammonia [33]. The same precursor was utilized by Gregory et al. to prepare gram quantities of copper nitride for powder neutron diffraction studies [34]. A contrasting approach was taken by Zachwieja and Jacobs in 1990 who used nitrate amine “single source” precursors, [Cu(NH_3_)x]NO_3_ (2 ≤ x ≤ 3) to prepare Cu_3_N without the need for ammonia, via a direct thermal decomposition reaction [35]. More recently, however, there has been added interest in the synthesis of different types of Cu_3_N nanostructures [36,37]. A flurry of different synthesis routes was applied to synthesise nitride nanoparticles, for example by solvothermal methods using copper azide in toluene or THF at moderate temperature [38], by thermal decomposition of Cu(NO_3_)_2_ in solutions containing long chain amines as solvents and capping agents [39,40,41], via the reaction of copper(II) acetate with NH_3_ gas in long-chain alcohol solvents [42] by a one-step process in which copper(II) methoxide reacts with benzylamine [43] or by reduction of Cu(II) to Cu(I) combined with nitridation of Cu(I) by hexamethylenetetramine [44].

Urea (CO(NH_2_)_2_) has also been investigated as an alternative—nongaseous—nitrogen source to NH_3_ [45]. In this work, we utilized CO(NH_2_)_2_ for Cu_3_N fabrication in a two-step process. Urea is soluble in water and its well-known hydrolysis process results in ammonia and carbon dioxide evolution. The thermally induced decomposition of urea in solution causes a relatively slow increase in pH which can be utilized to fabricate nanoparticles [46]. Urea can also play the role of capping and reducing agent in the synthesis of nanoparticles [47]. Moreover, utilizing CO(NH_2_)_2_ combined with ethylene glycol (EG) can improve the uniformity of the dispersed precipitate [48].

Generally, the development of facile, inexpensive synthetic methods is especially valuable given the emerging commercial applications of nanomaterials. Synthesis using long-chain amines [39,40,41] is becoming the most-utilized chemical method for the fabrication of Cu_3_N nanopowders. Implementing this route on a commercial basis is challenging, however, due both to the costs of the required chemicals and to the limits of morphological control that can be exerted [40]. In our approach, we propose important modifications to the classical approach to late transition metal nitride synthesis; namely the reaction of a suitable precursor in gaseous ammonia. Importantly, ammonolysis does not change the initial shape of the nanoparticles and so by using prescribed precursor morphologies, it is possible to exert intricate control over the copper nitride nanostructure. Moreover, ammonolysis can be applied to composite materials including substrates or matrices that are resistant to interaction with NH_3_ at temperatures of approximately 300 °C or less. We reveal experimental results here that demonstrate that exploiting oxygen-containing inorganic precursors, such as CuO and Cu(OH)_2_ in ammonolysis reactions, leads to the formation of single-phase Cu_3_N under a range of experimental conditions. Furthermore, Cu_3_N composites can be produced in the same way. A range of such experiments illustrates the diversity of Cu_3_N-based nanomaterials that can be fabricated by this precise two-step approach. In each case, the desired morphology is prescribed by the precursor design in step 1. Simple heating in ammonia then yields Cu_3_N pseudomorphically in step 2.

In this paper, as an example of this approach, copper(II) chloride dihydrate was used as a precursor for the fabrication of copper(II) oxide and copper(II) hydroxide nanoarchitectures (via synthesis of the hydroxide chloride, Cu_2_(OH)_3_Cl). The preparation of copper nitride micro- and nanostructures was then realized by ammonolysing the Cu(OH)_2_ and CuO nanoparticle precursors. Cu_3_N/SiO_2_ nanocomposites comprising Cu_3_N nanoparticles supported on silica could be assembled using the same precursors in closely related protocols. This is the first time that systematic studies of supported and unsupported Cu_3_N nanostructures synthesized by this method have been performed. As we demonstrate, the synthetic method can be applied to the growth of different shapes and sizes of nanocrystalline copper nitride, which, despite evidence of negligible porosity when unsupported, can nevertheless display specific surface areas of up to 45 m^2^ g^−1^.

## 2. Results and Discussion

### 2.1. Synthesis and Ammonolysis Reactions

In a typical synthesis, copper(II) chloride CuCl_2_·2H_2_O (3 mmol) was dissolved in a water/EG mixture (28 cm^3^; *v*/*v*, 1:8.3) in a round-bottomed flask to which CO(NH_2_)_2_ (17 mmol) was added with constant stirring. The resultant light green solution was heated to 95 °C over a period of 1.5–2.5 h. During the reaction, a green precipitate of Cu_2_(OH)_3_Cl formed from a deep blue solution. The final solid product was collected by centrifuging and was washed with water and alcohol several times before dispersing in acetone. A jade-green precipitate of Cu_2_(OH)_3_Cl was obtained from the remaining deep navy blue solution after leaving to stand at ambient temperature in air for several days.

**Figure 1:**
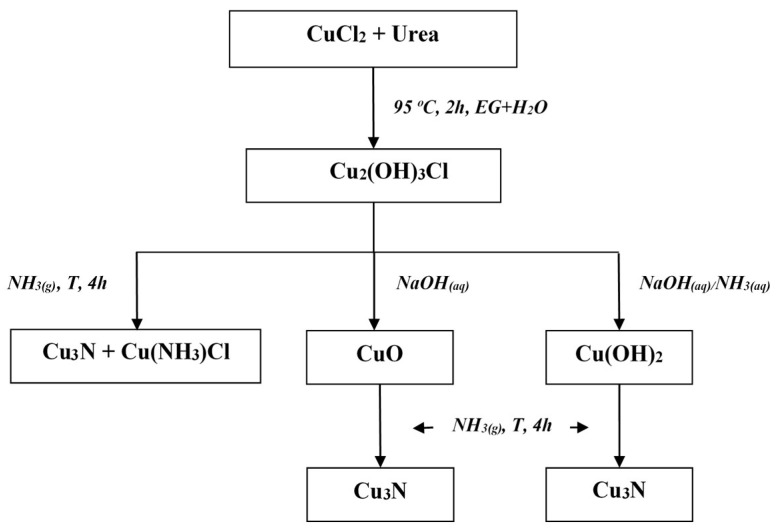
Schematic representation of the preparation processes for the respective powders.

To prepare CuO powder, a 0.01 M solution of NaOH was added dropwise into the previously described green Cu_2_(OH)_3_Cl powders dispersed in EtOH with constant stirring until a black precipitate formed. A very similar method was used to prepare Cu(OH)_2_ powder, with the exception that a few drops of NH_3_ (*aq*) were added to the Cu_2_Cl(OH)_3_ water dispersion prior to adding the 0.01 M NaOH solution. Both the oxide and hydroxide products were centrifuged, washed and then dispersed in acetone. The synthesis route is shown schematically in Figure 1.

As-received SiO_2_ aerogel is highly hydrophilic, with multiple -OH groups on the surface. The treatment of initial SiO_2_ with trimethylsilyl chloride was done as described in literature to completely remove the hydrophilicity on the surface [49,50,51]. The Cu_3_N/SiO_2_ composite preparation procedure was similarly congruent to the synthesis of the unsupported Cu_3_N nanoparticles by Cu(OH)_2_ powder and resulted in a final Cu_3_N loading of 3.3 or 5.4 wt.% on the SiO_2_ support. The Cu: urea molar ratio used in this process was 1:5. For a lower final load, the mixture of the CuCl_2_·2H_2_O salt, urea and SiO_2_ in H_2_O was stirred for 2 h at room temperature before heating. A few drops of 0.5 M NH_3_ (*aq*) were then added prior to adding NaOH dropwise until a blue solution was obtained. The resulting suspension was centrifuged, washed with deionized water and acetone before dispersing in acetone.

The ammonolysis experiments were performed in a horizontal tube furnace using NH_3_ gas (BOC, 99.98%) as the nitriding agent. In each reaction, the dry precursor powder was placed in a ceramic boat (60 mm × 20 mm × 20 mm) and transferred to the centre of the furnace. Reaction temperatures between 240–310 °C were selected (see below) and in all cases, the maximum temperatures were reached in ~45 min. The furnace was allowed to cool naturally under flowing NH_3_.

### 2.2. Characterization and Ammonolysis Reaction of Copper(II) Hydroxide Chloride Nanoparticles

**Figure 2:**
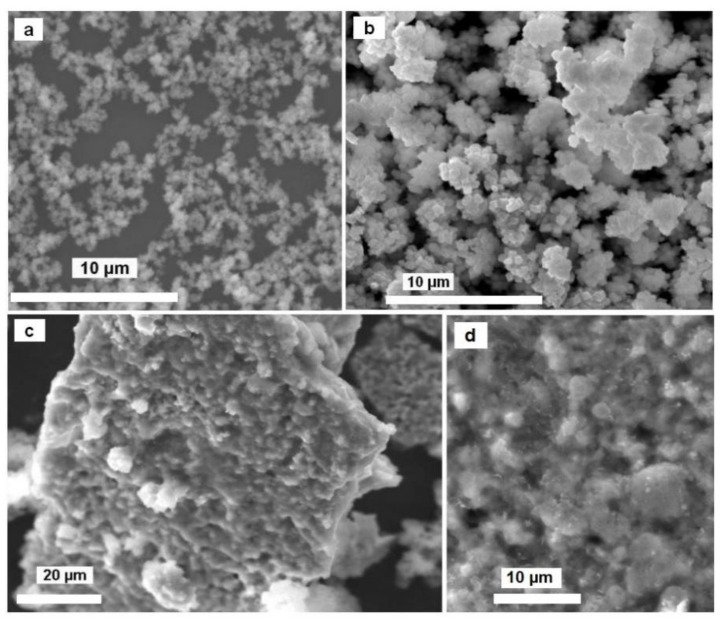
SEM images of as-prepared precursor powders: (**a**) sample **A**, (**b**) sample **B** and (**c**,**d**) sample **A** after ammonolysis reaction (4 h, 300 °C sample **1b**).

In the first step of our investigation, simple copper chloride nanoparticles were synthesized under various conditions. A SEM image of the powders obtained with CuCl_2_·H_2_O/CO(NH_2_)_2_ and GE/H_2_O molar ratios of 1:6 and 8.3:1 respectively (**A**) is presented in Figure 2a. In the case of **A**, a fine powder was obtained. The morphology and composition of the products were not affected by the gaseous environment (air or argon) in which the reactions were performed. SEM images for powder **B** fabricated under different conditions (using CuCl_2_·H_2_O/CO(NH_2_)_2_ and GE/H_2_O molar ratios of 1:5 and 6.7:1, respectively) revealed that the prepared particles take a cubic form with dimensions of ca. 300–500 nm across (Figure 2b). 

**Figure 3:**
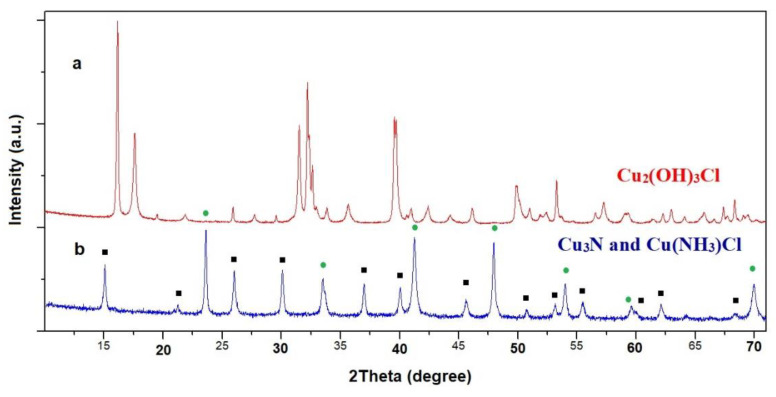
PXD patterns of (**a**) sample **A** as prepared and (**b**) sample **A** after heating in NH_3_ atmosphere at 300 °C (**1b**). Sample **A** could be indexed as single-phase atacamite, while peaks in the pattern from sample **1b** could be identified as Cu_3_N (■) and Cu(NH_3_)Cl (●).

The cubes form large spatial systems. Results from PXD and FT-IR experiments for the as-prepared samples (**A** and **B**) indicated the presence of Cu_2_(OH)_3_Cl. According to the literature, natural copper(II) hydroxide chloride, Cu_2_(OH)_3_Cl, exists in nature in four different mineral phases: atacamite, botallackite, paratacamite, and clinoatacamite [52,53]. Figure 3a shows a PXD pattern of the formed product (sample **A**). All the diffraction peaks could be indexed according to the orthorhombic atacamite structure. For **A**, indexing yielded a unit cell in the space group P*nma* with lattice parameters of *a* = 6.030(2) Å, *b* = 9.120(2) Å, *c* = 6.865(2) Å, which are in good agreement with the reported literature values [54].

The solution synthesis of Cu_2_(OH)_3_Cl powders has been previously described using NaOH/H_2_O_2_ [55], (NH_4_)_2_CO_3_ [56] or CO(NH_2_)_2_ [57]. In the last of these alternatives, the formation of Cu_2_(OH)_3_Cl from the hydrothermal reaction of copper(II) chloride and urea involves a hydrolysis−precipitation process. Urea is unstable under heating in aqueous solution and slowly liberates OH^−^ which reacts with Cu^2+^ and Cl^−^ in the reaction vessel to yield a green precipitate according to reactions:

CO(NH_2_)_2_ + 3H_2_O → 2OH^−^ + 2NH_4_^+^ + CO_2_, (1)

2Cu^2+^ + 3OH^−^ + Cl^−^ → Cu_2_(OH)_3_Cl. (2)

Ammonolysis experiments using **A** as a precursor were performed at three different reaction temperatures (240, 300 and 330 °C) for 4 h. The SEM images of the products heated under NH_3_ reveal that the initial particle shape was not completely preserved, with clusters of nanoparticles aggregating to form semiporous “cake-like” structures (Figure 2c). For each reaction temperature used, the composition of the product was identical and according to PXD data, a two-phase product consisting of Cu_3_N and Cu(NH_3_)Cl (Figure 3b) was obtained [58]. Phase fractions (wt%) were estimated using profile fitting within the PowderCell software package [59] and, as an example, a sample heated at 330 °C contained approximately 41% Cu_3_N and 59% Cu(NH_3_)Cl. IR measurements supported this analysis, with spectra clearly demonstrating the presence of N−H stretching and bending bands from the copper(I) monoamine phase at 3295, 3241 cm^−1^ (ν_as_NH), 3160 cm^−1^ (ν_s_NH), 1594 cm^−1^ (δ_a_(NH_3_)) and 1240 cm^−1^ (δ_s_(NH_3_)) [60]. The presence of copper nitride was also confirmed by a broad IR band at 664 cm^−1^ [61].

### 2.3. Characterization and Ammonolysis Reaction of Copper(II) Oxide and Copper(II) Hydroxide Nanoparticles

**Figure 4:**
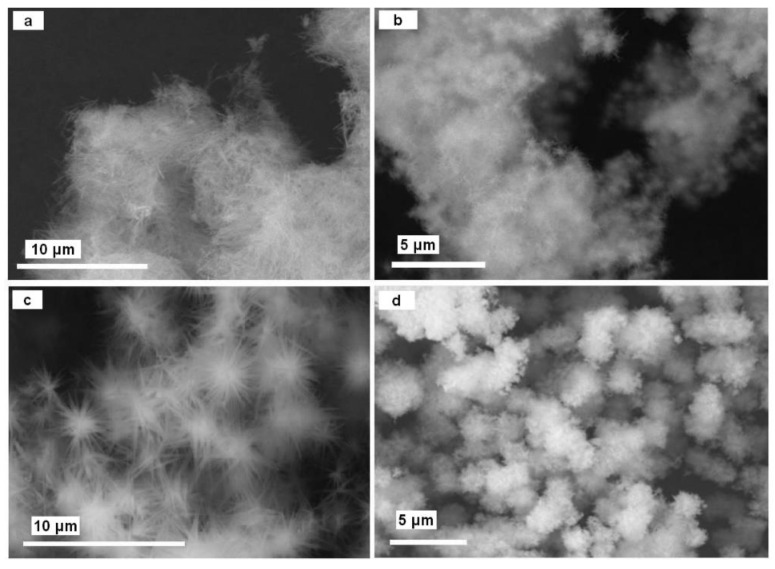
SEM images of powders: obtained by NaOH/NH_3_(*aq*) treatment of the Cu_2_(OH)_3_Cl (sample **A**) (**a**,**b**) and Cu_2_(OH)_3_Cl (sample **B**) (**c**) powders; obtained by NaOH treatment of the Cu_2_(OH)_3_Cl (sample **B**) powder (**d**).

As shown in the scheme in Figure 1, the reaction of Cu_2_(OH)_3_Cl with NaOH resulted in copper(II) oxide, which was obtained as a fine black powder product. The further addition of NH_3_(*aq*) led to the transformation of the initial fine powder to the nanoneedles or star-like particles of Cu(OH)_2_ (Figure 4a–c), depending on the reaction conditions. The identities of the synthesized CuO (see Figure 4d) and Cu(OH)_2_ powders were confirmed by PXD; all the diffraction peaks in the patterns could be assigned to either CuO (ICDD PDF card No. 05-0661) or Cu(OH)_2_ (ICDD PDF card No. 72-0140), respectively, and no impurity peaks were observed in either case. A mechanism for the conversion of copper(II) chloride in 0.01 M NaOH has been previously suggested in the literature [62]. SEM analysis in this previous case indicated an initial “etching” of the crystals of the starting material in the basic solution. The growth of needle-like structures on the crystalline surface was observed as the etching continued. The previous authors assumed a mechanism in which NaOH reacts with Cu_2_(OH)_3_Cl crystals and Cl^−^ is replaced at the interface with OH^−^ (Equation (3)). Strong deformations of the internal bulk structures occur as a result. Copper(II) hydroxide is known to form needle-like crystals in aqueous solution and “sisal-like” structures subsequently grow along the radical direction.

Cu_2_(OH)_3_Cl + OH^−^ → 2Cu(OH)_2_ + Cl^−^ (3)

In previous studies, CuO formation was realized by thermal treatment of the hydroxide. In our experiment copper(II) oxide occurred directly in solution, probably due to the synergistic effect of using ethanol as a solvent combined with a concentration of NaOH ten times less (influencing the pH accordingly) [63]. The solubility of CO(OH)_2_ and CuO in the ethanol-water system should also be considered and in the case of aqueous solutions, this difference is relatively large; the solubility of Cu(OH)_2_ = 1.3 × 10^−5^ mol L^−1^ as compared to 2 × 10^−7^ mol L^−1^ for CuO [64].

**Figure 5:**
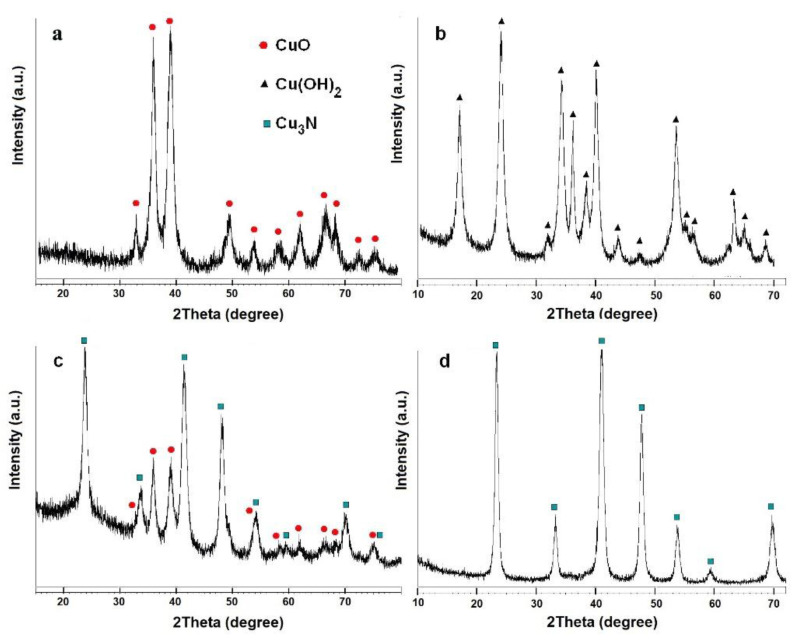
X-ray diffraction patterns of: (**a**) CuO, (**b**) Cu(OH)_2_ powders as-made and (**c**) CuO (**2a**), (**d**) Cu(OH)_2_ (**3b**) following ammonolysis. Red circles indicate CuO, black triangles indicate Cu(OH)_2_ and black squares represent Cu_3_N.

**Figure 6:**
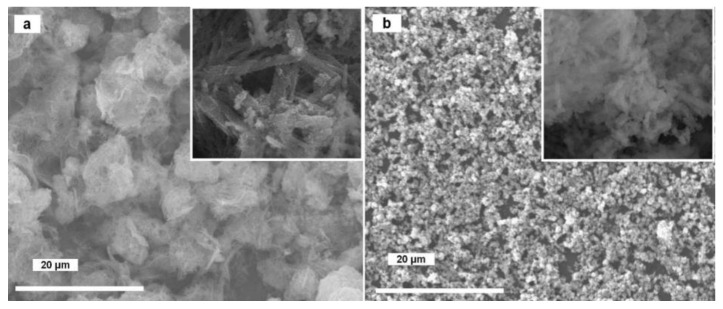
SEM images of as-prepared Cu_3_N powders: (**a**) sample (**2b**) (CuO) and (**b**) sample (**3b**) (Cu(OH)_2_) after ammonolysis (4 h, 300 °C). The inset shows a view of the samples magnified 150,000×.

The oxide and hydroxide samples were then heated under gaseous ammonia and the ensuing products were characterized by PXD (Figure 5), SEM (Figure 6) and BET. According to these experiments, the optimal conditions for the synthesis of phase-pure copper nitride are to use a temperature of 300 °C and a heating time of 300 min (Table 1).

Perhaps surprisingly, some experiments resulted in three-phase compositions (e.g., sample **4**), with the presence of all three common copper oxidation states (+2, +1 and 0; resulting from dehydration, nitridation and complete reduction, respectively). It is interesting to note that applying the selected precursors under the prescribed conditions led to pseudomorphic transformations from each precursor to copper nitride (Figure 6a,b).

Nitrogen adsorption−desorption experiments performed on different copper nitride samples commonly yield type II isotherms, which are typical for nonporous solids [65]. These adsorption isotherms are essentially linear in the range of P/P_o_ = 0–0.7 and condensation adsorption emerges in the region starting from P/P_o_~0.8 to 1.0. The specific surface area for the samples obtained from the Cu(OH)_2_ precursor shows a value of approximately 45 m^2^ g^−1^.

The total pore volume, however, was observed to be as low as ~0.15 cm^3^ g^−1^, essentially confirming that the material was nonporous. In the case of Cu_3_N fabricated from a copper oxide precursor, the BET surface area is lower and is manifestly dependent on the precursor synthesis route; CuO powders synthesized from **A** and **B** in turn produced copper nitride materials with specific surface areas of 37 m^2^ g^−1^ and 4 m^2^ g^−1^, respectively. These materials were also nonporous.

### 2.4. Synthesis of Cu_3_N/SiO_2_ Composites

Nanoparticles of binary inorganic copper compounds can provide a very interesting alternative to homogeneous copper complex catalysts for the cycloaddition of terminal alkynes and azides (such as the Huisgen 1,3-dipolar cycloaddition; HDC) and fulfil the requirements of a “click reaction” [66]. With such applications in mind, we were interested in exploring the precursor routes used here in the synthesis of supported Cu_3_N nanomaterials. To this end, we utilized copper(II) chloride as a precursor for the preparation of copper nitride supported on silica, Cu_3_N/SiO_2_. To achieve this, we modified the Cu_3_N synthesis process such that Cu_3_N/SiO_2_ nanocomposites could be achieved in a series of simple steps comprising: (i) silica modification; (ii) incorporation of Cu_2_(OH)_3_Cl into silica; (iii) conversion of Cu_2_(OH)_3_Cl + silica into a Cu(OH)_2_/SiO_2_ nanocomposite and (iv) ammonolysis of the Cu(OH)_2_/SiO_2_ precursor to yield Cu_3_N/SiO_2_.

**Figure 7:**
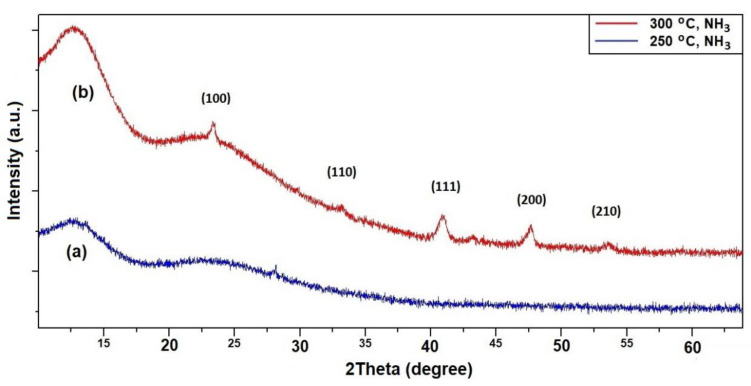
PXD patterns of Cu(OH)_2_/SiO_2_ composites heated under flowing NH_3_ gas at: (**a**) 250 °C (blue) and (**b**) 300 °C (red). The Miller indices (hkl) for copper(I) nitride, Cu_3_N, are indicated.

The Cu(OH)_2_/SiO_2_ nanocomposites were prepared as detailed in the experimental section. Ammonolysis reactions using the Cu(OH)_2_/SiO_2_ precursor were performed at 250, 300 and 310 °C (over a period of 4–5 h). After heating under gaseous NH_3_, the initially blue powders became either grey or light red in colour. Despite the colour change, only amorphous-appearing PXD patterns were observed for, e.g., a sample heated for 4 h at 250 °C (Figure 7a). This may suggest that the Cu_3_N particles are of submicron dimensions and/or possess no long-range structural order, hence producing no Bragg reflections. Alternatively, the low wt.% loading of Cu_3_N particles might be constricted inside the internal porosity of the SiO_2_ matrix (support), rendering them undetectable in the X-ray diffraction patterns.

On ammoniating a nanocomposite sample at 310 °C, PXD showed that the product was the intended Cu_3_N/SiO_2_ material (Figure 7b). Only two examples of Cu_3_N/SiO_2_ materials exist in the literature [67,68]. The impregnation of SiO_2_ by a Cu(II) salt in solution followed by deposition–precipitation is an effective way to fabricate oxidic copper-based SiO_2_ composites [69,70]. We have shown that such combined impregnation−deposition–precipitation approaches work equally well for nitride-SiO_2_ composites. Our early-stage observations are currently being followed by wider, systematic studies of the ammonolysis of supported materials, which will be reported in due course.

**Figure 8:**
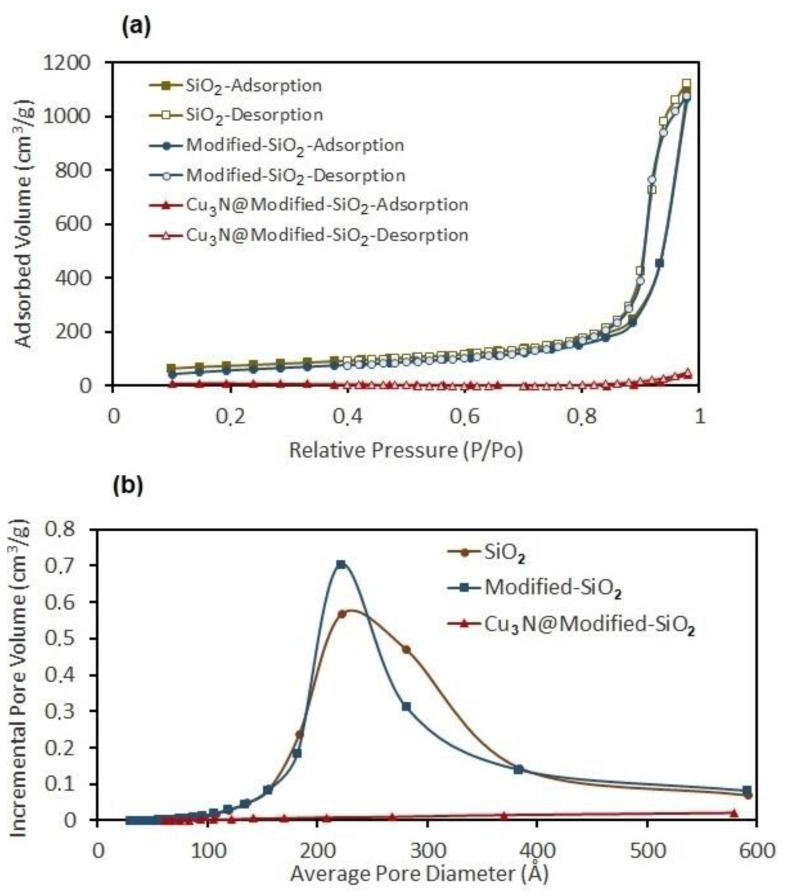
(**a**) BET isotherms and (**b**) the incremental pore volumes registered for the as-received SiO_2_ aerogel, modified SiO_2_ aerogel, and Cu_3_N/modified SiO_2_ aerogel samples.

It is useful to consider the absorption behavior of both the silica host and the Cu_3_N/SiO_2_ nanocomposite and the resulting implications for the specific surface area of the materials (Figure 8a,b). The pristine SiO_2_ aerogel exhibits a total adsorption volume of 1125.054 cm^3^ g^−1^. Once modified (with trimethyl silyl chloride) the total adsorption of the silica dropped slightly to 1075.616 cm^3^ g^−1^. Although this constituted a 4.40% drop in the total sorption capacity, there was otherwise little difference in the adsorption behavior (as perceived from the nitrogen sorption isotherms) of the silica before and after treatment with trimethyl silyl chloride. There were fundamental changes in the internal porosity, however, and when considering the incremental pore volume (Figure 8b), the pore size distribution became narrower and sharper after the treatment (but otherwise remained in the mesoporous range).

By comparison, the Cu_3_N/SiO_2_ nanocomposite had a very limited nitrogen adsorption ability, with a total adsorption of 49.345 cm^3^ g^−1^. This constitutes a 95.41% drop in the total nitrogen sorption and hence, in relative terms, the pore volume almost disappears. This could be a reflection of the fact that the Cu_3_N nanoparticles were confined within the modified-SiO_2_ aerogel, with uniform particle size of the same order of magnitude as the pore size of the host—approximately 21–22 nm.

## 3. Materials and Methods

### 3.1. Starting Materials

Copper(II) chloride dihydrate, sodium hydroxide, urea, ammonium hydroxide solution (25% NH_3_ in H_2_O) and ethylene glycol (EG) were used as received (Sigma-Aldrich, St. Louis, MO, USA). The silica support material, Aerosil-200 was provided by Evonik free of charge. The silica was modified prior to use by treating with trimethyl silyl chloride (Sigma-Aldrich, St. Louis, MO, USA).

### 3.2. Materials Characterization

All products were characterized by powder X-ray diffraction (PXD) using either a Philips XPERT Pro θ-2θ (Malvern Panalytical Ltd., Malvern, UK) or a Bruker D8 diffractometer (Bruker AXS, Karlsruhe, Germany) with CuKα or CuKα_1_ radiation, respectively. Whereas PXD data for the final products were collected directly from powders, PXD data for the precursors were collected from samples dispersed in acetone dropped onto the PXD sample holder. Phase identification was performed by search-match procedures with access to the International Centre for Diffraction Data (ICDD) powder diffraction file (PDF) and by comparison to patterns generated from Inorganic Crystal Structure Database (ICSD) data using PowderCell v.2.3 [59]. Scanning electron microscopy (SEM) was performed by using three instruments: first, a Philips XL 30 environmental (E)SEM equipped with an Oxford Instruments INCA Energy 250 energy dispersive X-ray (EDX) spectrometer (EHT = 20 kV, spot size 5), second, a LEO 1430 VP (LEO Electron Microscopy Ltd., Cambridge, UK) microscope equipped with a Quantax 200 (XFlash 4010 detector, Bruker AXS, Karlsruhe, Germany) EDX spectrometer (HV mode, SE, EHT = 10–20 kV, beam current 100 μA), and finally a Quanta 3D FEG instrument (FEI, Hillsboro, OR, USA) (EHT = 30 kV). The samples were imaged without coating and were placed onto carbon tabs fixed to aluminium SEM stubs. Qualitative analysis of bonding was performed by Fourier transform infrared spectroscopy (FTIR; Shimadzu 8400S spectrometer, Shimadzu Corp., Kyoto, Japan). The surface areas of the synthesized powders were determined by applying BET analysis to nitrogen adsorption−desorption data, which were registered at liquid nitrogen temperature using a Micromeritics Gemini instrument (Norcross, GA, USA).

## 4. Conclusions

These studies demonstrate that copper nitride powders can be successfully synthesized from a range of oxygen-containing inorganic precursors. With the correct choice of experimental parameters, nitridation is favored over simple reduction. The choice of precursor and synthesis conditions enables the size and morphology of the nitride micro- and nanoparticles to be manipulated. Each of the precursors—Cu_2_(OH)_3_Cl, Cu(OH)_2_ and CuO—can be prepared from a copper(II) chloride starting material by urea hydrolysis in an ethylene glycol/water medium below 100 °C. The first of these precursors, Cu_2_(OH)_3_Cl, has limited utility in the preparation of Cu_3_N, however, since not only is the ammonolysis process not pseudomorphic (thus offering little morphology control), but it also proves challenging to prepare Cu_3_N without the presence of the copper(I) monoamine, Cu(NH_3_)Cl.

Alternatively, the Cu_2_(OH)_3_Cl precursor can be converted to Cu(OH)_2_ or CuO *in solution* by the addition of NaOH/NH_4_OH. The advantage of these solution syntheses is that CuO nanoparticles can be obtained quickly and without heating, retaining a similar morphology to the copper(II) chloride starting material. Similarly, nanostructured Cu(OH)_2_ can be obtained from the chloride in solution, forming as needles on the nanoscale. Both CuO and Cu(OH)_2_ precursors are successful in producing single-phase Cu_3_N under a range of experimental conditions. At an elevated temperature (of ca. 330 °C and above), the nitride seemingly decomposes, with copper metal as the predominant product. Preliminary observations suggest that mesoporous silica can act as an efficient support for Cu_3_N. Importantly, modification with trimethylsilyl chloride removes the hydrophilicity from the surface of SiO_2_ aerogel to enable successful ammoniation of Cu(OH)_2_/SiO_2_ composites. Thus, we successfully synthesized Cu_3_N/modified-SiO_2_-aerogel nanocomposites and the hydrolysis decomposition of Cu_3_N by solid-state acidic media was completely excluded.

## Figures and Tables

**Table 1:** Experimental conditions for samples prepared by ammonolysis reaction.

Sample No.	Precursor	Temperature/°C	Time/min	Product Phases from PXD (wt%) *
**1a**	Cu_2_(OH)_3_Cl	240	240	Cu_3_N (38) > Cu(NH_3_)Cl (62)
**1b**	Cu_2_(OH)_3_Cl	300	240	Cu_3_N > Cu(NH_3_)Cl
**1c**	Cu_2_(OH)_3_Cl	330	240	Cu_3_N (41) > Cu(NH_3_)Cl (59)
**2a**	CuO	280	180	Cu_3_N (69) > CuO (31)
**2b**	CuO	310	300	**Cu_3_N**
**2c**	CuO	330	240	Cu_3_N > Cu
**3a**	Cu(OH)_2_	300	240	Cu_3_N (77) > CuO (23)
**3b**	Cu(OH)_2_	310	300	**Cu_3_N**
**3c**	Cu(OH)_2_	330	240	Cu_3_N > Cu
**4**	Cu(OH)_2_	310	300	Cu_3_N (55)/CuO (33)/Cu (12)

* Phase fractions (wt%) were estimated using profile fitting within the PowderCell software [59]. For some samples the phase composition was not estimated due to the low peak intensity of the corresponding XRD patterns.

## Data Availability

Data is contained within the article.

## Sample Availability

The samples are not available from the authors.
